# Endoscopic closure of an esophago-bronchial fistula after esophageal atresia repair using autologous adipose stromal vascular fraction and platelet-rich plasma injection

**DOI:** 10.1055/a-2905-4711

**Published:** 2026-08-21

**Authors:** Roos E. Pouw, Joris A. Van Dongen, Ludovica Magni, Arnold J. Bittermann, Sander Zwaveling, Michiel P. van Wijk, Stefaan H. A. J. Tytgat

**Affiliations:** 1 Department of Gastroenterology and Hepatology 8124 UMC UtrechtUtrechtThe Netherlands; 2 Department of Plastic-, Reconstructive- and Hand Surgery 8124 UMC UtrechtUtrechtThe Netherlands; 3 Department of Pediatric SurgeryWilhelmina Childrenʼs Hospital, UTC UtrechtUtrechtThe Netherlands; 4 Department of Pediatric OtorhinolaryngologyWilhelmina Childrenʼs Hospital, UMC UtrechtUtrechtThe Netherlands; 5 Department of Pediatric SurgeryEmma Children's Hospital, Amsterdam University Medical CenterAmsterdamThe Netherlands; 6 Department of Pediatric GastroenterologyEmma Childrenʼs Hospital, Amsterdam University Medical Center, Location Vrije UniversiteitAmsterdamThe Netherlands; 7 Amsterdam Gastroenterology Endocrinology Metabolism and Amsterdam Reproduction and Development Research InstitutesAmsterdam UMC, VU UniversityAmsterdamThe Netherlands

Esophageal–airway fistulas are severe complications after esophageal atresia (EA) repair, occurring in 2–14% of patients and associated with chronic aspiration, recurrent respiratory infections, feeding difficulties, and failure to thrive. Endoscopic closure is less invasive but has limited success, whereas surgical revision is more effective yet carries substantial morbidity 1 . Regenerative therapies, including autologous adipose stromal vascular fraction (SVF) and platelet-rich plasma (PRP), have shown promising results in adult fistula treatment 2 3 .

**Fig. 1:**
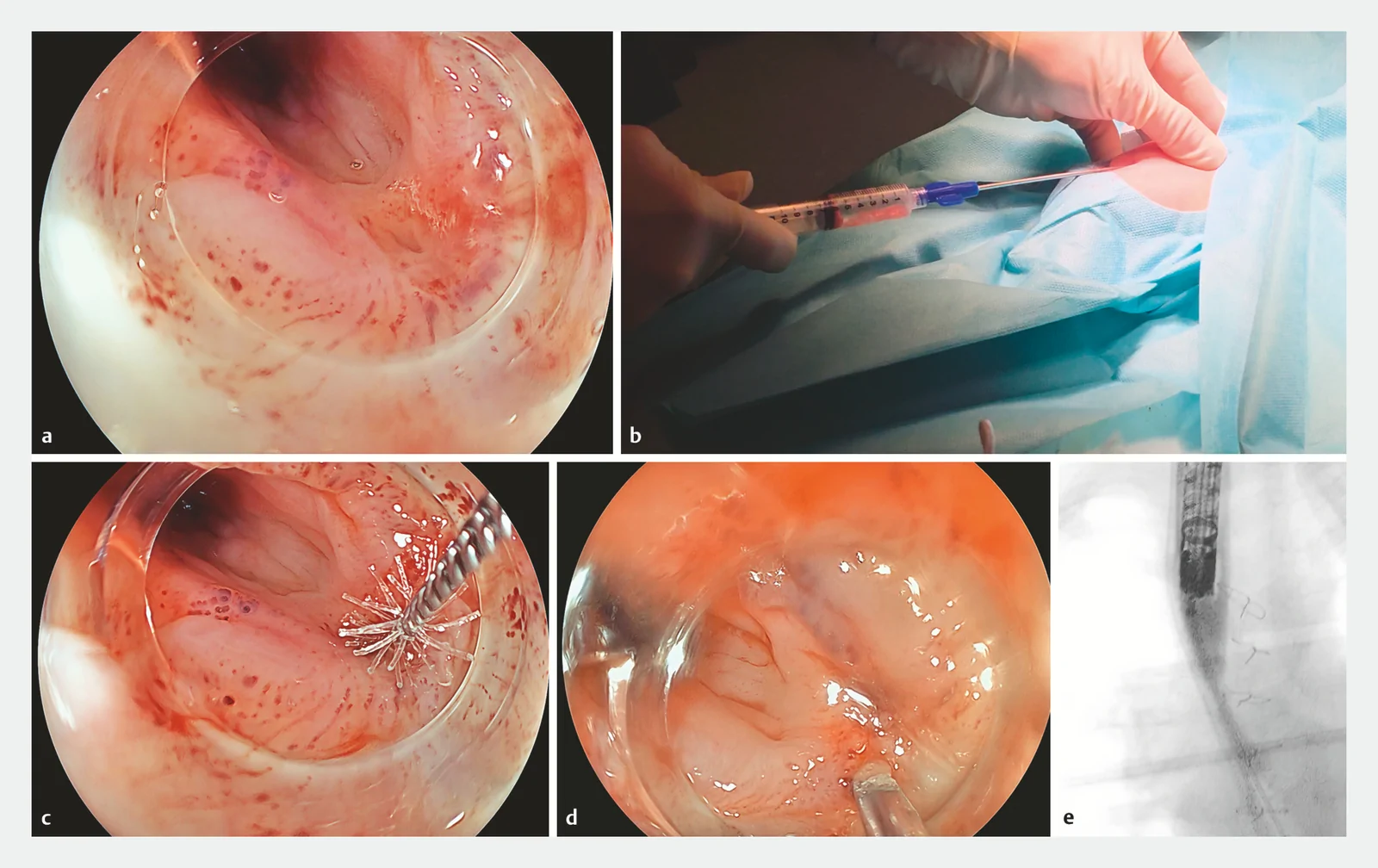
**a** During endoscopy the bronchoesophageal fistula is identified. **b** Adipose tissue is harvested from the upper legs. **c** The fistula is mechanically debrided using a cytology brush. **d** The SVF/PRP mixture is injected around the fistula in the submucosa. **e** Closure of the fistula was confirmed by contrast infusion of the esophagus after distal occlusion of the esophagus with a Fogarty catheter.

A 16-month-old boy with long-gap type-C EA and tracheomalacia developed anastomotic leakage and a refractory stricture. Surgical revision was complicated by leakage and the formation of a cavernous esophagobronchial fistula to the right upper lung ( Fig. 1 **a** ). Endotracheal closure was not feasible, and endoscopic esophageal brush debridement was unsuccessful. Further surgery was considered undesirable, and the patient’s condition deteriorated with recurrent respiratory infections and severe failure to thrive.

Endoscopic fistula closure using autologous SVF/PRP was performed ( Video 1 ). Adipose tissue was harvested ( Fig. 1 **b** ) and mechanically fractionated and centrifuged to obtain SVF (Arthrex ACP-SVF kit 4 ), and PRP was prepared from peripheral blood. After fistula identification and tract brushing ( Fig. 1 **c** ), SVF/PRP was injected circumferentially around the esophageal fistula orifice using a 7.9-mm endoscope (Fujifilm; Fig. 1 **d** ).

The video demonstrates the identification of the fistula; the preparation of the adipose stromal vascular fraction (SVF) and platelet rich plasma (PRP); the treatment of the fistula; and the mechanistical background information and endoscopic follow-up.Video 1

Following the procedure, respiratory symptoms resolved, infections ceased, and the child gained 1 kg. At 8 weeks, endoscopy with fluoroscopy confirmed fistula closure ( Fig. 1 **e** ).

SVF contains adipose stromal cells, endothelial progenitors, and immune cells, which exert anti-inflammatory and pro-regenerative effects. These effects are enhanced by the extracellular matrix, providing a perivascular niche for cell retention and sustained paracrine factor release. PRP further augments regenerative signaling. Mechanical shear stress during SVF isolation activates stromal cells toward a pro-regenerative phenotype, supporting SVF/PRP as a low-risk strategy for inoperable pediatric esophageal–airway fistulas 5 . To our knowledge, this represents the first reported infant successfully treated with endoscopic autologous adipose SVF/PRP for esophagobronchial fistula closure after EA repair.

Endoscopy_UCTN_Code_TTT_1AO_2AI

Citation Format

Endoscopy 2026; 58: E525–E526. DOI: 10.1055/a-2846-4773
